# Antioxidants Potential of the Filamentous Fungi (*Mucor circinelloides*)

**DOI:** 10.3390/nu9101101

**Published:** 2017-10-07

**Authors:** Ahsan Hameed, Syed Ammar Hussain, Junhuan Yang, Muhammad Umair Ijaz, Qing Liu, Hafiz Ansar Rasul Suleria, Yuanda Song

**Affiliations:** 1 Colin Ratledge Center for Microbial Lipids, School of Agriculture Engineering and Food Science, Shandong University of Technology, Zibo 255049, China; ahsanhameed@outlook.com (A.H.); ammarshah88@yahoo.com (S.A.H.); judywoniu@yahoo.com (J.Y.); qingliu0906@yahoo.com (Q.L.); 2 Key Laboratory of Meat Processing & Quality Control, College of Food Sciences, Nanjing Agriculture University, Nanjing 210095, China; mumairb@outlook.com; 3 UQ School of Medicine, University of Queensland, Brisbane, QLD 4072, Australia; hafiz.suleria@uqconnect.edu.au; 4 Department of Food, Nutrition, Dietetics & Health, Kansas State University, Manhattan, KS 66506, USA

**Keywords:** antioxidants, filamentous fungi, *Mucor circinelloides*, food microbiology, natural antioxidants

## Abstract

Three important strains of *Mucor circinelloides* grown in complete and minimal media for specified period (72 h, 120 h and 168 h) under submerged fermentation conditions were investigated for their potential antioxidants/secondary metabolite production. All mycelial extracts demonstrated effective antioxidant activities in terms of β-carotene/linoleic acid bleaching, radical scavenging, reduction of metal ions and chelating abilities against ferrous ions. Different extraction methods and solvent systems affected the recovery yield and antioxidant activities of the extracts significantly (*p* ≤ 0.05). Ethanolic extracts were found to be rich source of antioxidant components and subsequently more effective in antioxidant properties. Fermentation period and media used also significantly affected (*p* ≤ 0.05) the antioxidant production and the resulting antioxidant properties. The (ethanolic) extracts of all the strains from late exponential growth phase (120 h) showed highest antioxidant production with topmost reducing, chelating and radical scavenging capabilities. Strain MC277.49 was found to be the highest producer of antioxidants followed by MC108.16 and WJ11. Phenolic compounds were detected significantly in higher (*p* ≤ 0.05) amount succeeded by the condensed tannins and flavonoids. Total phenol content of each extract was attributed to overall antioxidant capacity. Submerged fermentation with nutritional stress conditions were found to be excellent way of producing surplus amount of natural antioxidants/secondary metabolites with their vast potential commercial application in food and pharmaceutical industries.

## 1. Introduction

Reactive oxygen species (ROSs), in high concentration, are considered a serious issue to human health as their overproduction can lead to rapid aging via DNA and protein damage, cardiovascular diseases, cancer, immune dysfunction, inflammation, renal failure and lipid oxidation [1]. To counteract these ROSs, living cells have natural antioxidant production system which is helpful in maintaining the redox status of body below the threshold level by controlling these ROSs. The loss of balance between the free radical production such as high ROSs production and loss of natural endogenous antioxidant production can lead to a serious health phenomenon called “oxidative stress”. In the case of failure to meet the required level of endogenous natural antioxidant production by living cells, this demand can be fulfilled by exogenous dietary natural antioxidant sources to avoid the oxidative stress and consequently above mentioned lethal ailments [2]. Food items such as fruits, vegetables, seeds, flowers, seafood, tea, herbs and spices are considered the traditional rich sources of antioxidants and their antioxidant potential has been thoroughly explored, stated and available in the literature [3,4,5]. Recent advancements in biotechnology, microbiology and progress in genetic research has made it easy for researchers to explore, detect and identify primary and secondary metabolites which has high antioxidant capacity, anticancer, anti-inflammatory and antibacterial characteristics from cheap and easily available non-traditional sources such as fungi.

Fungi are the most diverse and abundant group on the planet and are an excellent candidate for bioactive metabolite production due to their resemblance to the animal system. Recently, molecular screening methods claimed there are as many as 5.1 million species of fungi, of which only approximately 100,000 have been reported in literature [6]. Among these fungi, 14,000 species are mushrooms, 5000 are macro fungi and more than 1800 fungi have been identified as possessing pharmacological, therapeutic and medicinal features [6]. The diverse fungi kingdom is divided into various phyla such as Ascomycota (sac fungi), Basidiomycota (club fungi), Zygomycota, Blastocladiomycota and Chytridiomycota. Most of the pharmacological, therapeutic and medicinal investigations have focused only on the strains or species from Ascomycota and Basidiomycota [7,8], while Zygomycota is comparatively less investigated. Further, most of the members of the diverse, high antioxidant potential exhibiting, secondary metabolite rich endophytic fungi group also belong to Ascomycota and Basidiomycota divisions, while few identified members belong to genera *Mucor* and *Umbelopsis* of zygomycota [7]. Endophytic fungi play an important role in adaption and morphogenesis of host plant by defending it against insects, predators, microbial pathogens and latent pathogens. Similarly, order Mucorales of Zygomycota is considered a sister group of Entomophthorales (insect pathogens) [9]. The pathogenicity of Mucorales is largely believed to be due to endocellular excretions and production of subtilisins, chitinases, proteinases and antioxidant proteins (e.g., superoxide dismutase, catalase and peroxidase) [9].

From industrial point of view, the class Zygomycetes of Zygomycota is more important than its second class Trichomycetes because of its two widely used bioactive compound producing genera: *Mucor* and *Rhizopus.* In short, Zygomycetes can produce a wide range of metabolites including enzymes, lipids, ethanol, organic acids, food colorants, amino acids, chitosan, chitin and proteins [10]. Briefly, genus *Rhizopus,* specifically, has been majorly exploited to produce lactic acid, fumaric acid, amylases, pectinases, steroids, lipases, ureases, and tannases, whereas genus *Mucor* is considered a good source for cellulases, phytases, proteases, ethanol, lipids and food colorants [10,11]. Moreover, the production of these metabolites is not a normal physiological metabolism related phenomenon and generally requires nutritional stress (e.g., N or P limitation) to produce and store these aforementioned metabolites in mycelium [11]. Due to large storage capability of these metabolites in their mycelium/biomass as well as their nutritional and pharmaceutical importance, there is an increasing interest (in fact, it is in the practical stage now) in utilizing the biomass of zygomycetes as a source of microbial proteins, microbial lipids, microbial ethanol, microbial food colorants, and microbial bioactive components such as essential amino acids, antibiotics and chitosan in food, aquaculture feed and pharmaceutical industries [10].

To summarize the above discussion, we hypothesize that two genera of zygomycetes (*Mucor* and *Rhizopus*) can be a great source of natural antioxidants and these natural antioxidants can be termed as “microbial antioxidants” because of microbes/fungi being their source of origin. Moreover, the natural production of microbial antioxidants from understudy filamentous fungi is also advantageous in terms of being: (i) cost effective; (ii) time saving; (iii) easy to handle; and (iv) non-competitive with food sources. The former two qualities of microbial antioxidant production from *Mucor circinelloides* (*MC*) may be attributed to the “genetic richness” features of these filamentous fungi, as the biomass of *MC* can be easily generated in fermentation using several cheap carbon sources under controlled condition within preset time. The latter two benefits of production of microbial antioxidants spare us from the arduous work of cultivating the (fruit/flower) plants/crops/herbs in arable land for an entire season with pertaining concerns of climatic and environmental hazards. The traditional sources of natural antioxidants are human consumable (fruit/flower) plants/crops/herbs but commercial production of natural antioxidants from these traditional sources may increase food competition globally. The production of natural antioxidants from non-traditional sources including microbes/fungi may be an imitative/alternative strategy to deal with this global issue. Hence, to test our hypothesis, we investigated the antioxidant potential of important strains of both genera. Although some recent scientific reports illustrate the antioxidant potential of genus *Rhizopus* (e.g., *Rhizopus oryzae*) along with food used for fermentation [12], according to the best of our knowledge, the literature is still lacking data on the antioxidant potential of important strains of genus *Mucor*. Therefore, in this study, we investigated the antioxidant capacity of important strains of *Mucor circinelloides*.

Scientifically, *Mucor circinelloides* belongs to family *Mucoraceae*, order Mucorales, sub class *Incertae sedis* and class Zygomycetes. The three strains we investigated are *Mucor circinelloides f. lusitanicus* (CBS 277.49), *Mucor circinelloides* (WJ11) and *Mucor circinelloides* (CBS 108.16). These strains are excellent producers of β-carotene, lycopene, lipids and bioactive component e.g., γ-Linolenic acid (GLA) [13,14], and are also easy to handle and produce with availability of molecular and transformation tools and genome sequence features [14,15]. Consequently, the objectives of our investigation are to: (i) explore the antioxidant capacity of chosen strains; (ii) search the effects of complete media entitled Kendrick and Ratledge Media (K and R) and nitrogen (N) limited media called modified Kendrick and Ratledge Media (MKR) [16] on antioxidant potential; (iii) check the influence of fermentation period on antioxidant capacities; and (iv) find the best strain(s) for antioxidant production.

## 2. Materials and Methods

### 2.1. Chemicals, Solvents and Reagents

All chemicals used were of molecular biology/analytical grade or higher where appropriate. 2,2′-azino-bis (3-ethylbenzothiazoline-6-sulfonic acid) (ABTS), α-tocopherol, aluminum chloride (AlCl_3_), ammonium acetate, ammonium dihydrogen phosphate, ammonium sulfate, β-carotene, butylated hydroxytoluene (BHT), calcium carbonate, (+)-catechin, copper (II), d-(+)-glucose, ethyl acetate, ethanol, ethylenediaminetetraacetic acid (EDTA), ferrous chloride, ferrozine, Folin–Ciocalteu reagent, gallic acid, linoleic acid, neocuproine, phosphate buffer, potassium ferric chloride, potassium hydroxide, potassium persulfate, potassium phosphate (monobasic and dibasic), quercetin, saturated sodium carbonate, sodium chloride, sodium hydroxide, sodium phosphate (monobasic and dibasic), sodium sulfate, sulfuric acid, tannic acid, trichloroacetic acid (TCA), trolox, TWEEN 20, vanillic acid, yeast extract and vanillin were all supplied by Sigma-Aldrich (St. Louis, MO, USA).

### 2.2. Strains, Seed Preparation and Fermentation Conditions

*M. circinelloides* (WJ11) isolated from the soil at Jiangnan University was used in this study [14]. *M. circinelloides f. lusitanicus* (CBS 277.49) was kindly provided by Prof. Victoriano Garre, Department of Genetics and Microbiology, University de Murcia, Spain. *M. circinelloides* (CBS 108.16) was purchased from China General Microbiological Culture Collection Center, Institute of Microbiology, Chinese Academy of Sciences, Shanghai, P.R China. Aliquots (100 μL) of spore suspension (approximately 10^7^ spores/mL) from each strain (i.e., WJ11, CBS 277.49 and CBS 108.16) were inoculated into 150 mL Kendrick and Ratledge medium (K & R medium) held in 1 L flasks equipped with baffles to improve aeration. The K and R medium contained 30 g/L glucose, 3.3 g/L diammonium tartrate, 7.0 g/L KH_2_PO_4_, 2.0 g/L Na_2_HPO_4_, 1.5 g/L MgSO_4_·7H_2_O, 1.5 g/L yeast extract, 0.1 mg/L CaC1_2_·2H_2_O, 8 mg/L FeC1_3_·6H_2_O, 1 mg/L ZnSO_4_·7H_2_O, 0.1 mg/L CuSO_4_·5H_2_O, 0.1 mg/L CO (NO_3_)·6H_2_O and 0.1 mg/L MnSO_4_·5H_2_O, pH 6.0. Seed cultures were incubated for 24 h at 30 °C with shaking at 150 rpm and then used at 10% (*v*/*v*) to inoculate 2 L fermenters (Celligen 315, New Brunswick, Germany) containing 1.5 L K & R or modified K & R medium (2 g diammonium tartrate, 80 g glucose per liter plus inorganic salts) for intended duration, i.e., 3 (72 h), 5 (120 h) and 7 (168 h) days. The pH was maintained at 6.0 by auto-addition of 4 M KOH or 2 M H_2_SO_4_.

### 2.3. Preparation of Extracts

The mycelium was collected, freeze dried, ground and extracted according to the method as previously described by Smith et al. [17] with some minor modifications. Briefly, after the preset time of fermentation, i.e., 3, 5, and 7 days, the mycelium was harvested by filtration and washed three times with deionized water to remove any remaining media or mineral ions. After thorough washing and removing maximum remaining water, mycelia were freeze-dried using an Alpha 1–4 LD plus freeze drying unit (Labconco, Germany). The lyophilized mycelia were ground to a fine powder (1.0 mm (No. 10) mesh), which was served as dried extract (crude extract). The fine powder of lyophilized mycelia was further extracted using hot water or ethanol extraction processes for comparison of antioxidant activity as well as to check the effect of different extraction processes on antioxidant activity.

For the ethanol extract, freeze-dried mycelial biomass (5 g) was accurately weighed into a 100 mL sterilin tubes and shaken overnight (18–24 h) with 50 mL of ethanol at room temperature. The extract was then filtered through Whatman paper No. 1 (×2) by vacuum filtration. The residue was then resuspended in another 50 mL of ethanol. The process was repeated twice. The combined ethanol filtrate was transferred to a pre-weighed sterilin tube and the solvent was evaporated by applying a constant flow of nitrogen. Residual ethanol was removed by vacuum pressure at 30 °C overnight in an oven and the dry weight was recorded.

Hot water extraction was performed on Electro-thermostatic water bath (Model: DK-8D, Shanghai Samsung Laboratory Instrument Co. Ltd., Shanghai, China). Dry mycelial biomass (0.5 g) was accurately weighed into the 50 mL glass tubes. After the addition of 10 mL deionized water, the mixture was heated at 100 °C for 20 min under reflux and allowed to cool for 20 min. Afterwards, the filtrate was separated from the mycelial biomass by vacuum filtering using No. 42 (×2) Whatman filter paper. The process was repeated twice. The combined filtrate was then freeze-dried and the dry weight was recorded. Both dried extracts were used directly for analysis of antioxidant components or re-dissolved in ethanol or deionized water to a concentration of 100 mg/mL and then diluted to 50, 20, 10, 5, 1, 0.1 and 0.01 mg/mL for further use.

### 2.4. Determination of Antioxidant Components

#### 2.4.1. Total Phenolic Contents (TPC)

Total phenolic content of the fungal extracts was estimated using the Folin–Ciocalteu reagent method according to Suleria et al. [18] with minor modification. Sample extracts (250 µL) were mixed with 250 µL of 10% (*v*/*v*) Folin–Ciocalteu reagent, followed by the addition of 500 µL saturated sodium carbonate (10%, *w*/*v* aqueous solution) after 2 min of incubation at room temperature. The mixture was placed in the dark for 1 h. Absorbance was then measured at ƛ_750 nm_. The concentration of total phenols was calculated based on a calibration curve using Gallic acid ranging from 10 to 100 µg/mL. The phenol contents were expressed as gallic acid equivalent (GAE), which reflects the phenol content, as the amount of Gallic acid units in 1 g of extract (mg GAE/g).

#### 2.4.2. Total Flavonoids Contents (TFC)

Flavonoid contents were measured according to the AlCl_3_ method of Quettier-Deleu et al. [19]. Each extract (0.5 mL) at a concentration of 10 mg/mL was mixed with 1.0 mL of a 2% (*v*/*v*) AlCl_3_·6H_2_O ethanolic solution and the absorbance was measured 10 min later at ƛ_430 nm_. Quantification of flavonoids was calculated based on a calibration curve of quercetin (0.01–50 μg/mL) and is expressed as Quercetin equivalent (QE), which reflects the flavonoid content as the amount of Quercetin units in 1 g of extract (mg QE/g).

#### 2.4.3. Total Condensed Tannin (TCT)

Total condensed tannin contents were determined according to the method of Yim et al. [20]. Ethanolic and aqueous fungal extracts were dissolved in ethanol and water, respectively. An aliquot of each extract (50 µL) was mixed with 1.5 mL of 4% (*w*/*v*) vanillin in ethanol, followed by the addition of 750 µL concentrated HCl. After mixing well, the mixture was allowed to stand at room temperature for 20 min in darkness. The blank was 4% (*v*/*v*) concentrated HCl in ethanol. The absorbance was read against a corresponding blank at ƛ_500 nm_. (+)-Catechin was used to prepare the standard curve (50–1000 μg/ mL) and the results were expressed as catechin equivalent (CE), which reflects the tannin content as the amount of catechin in 1 g of extract (mg CE/g).

### 2.5. Antioxidant Assays

#### 2.5.1. Total Antioxidant Activity (TOAA) Determination by β-carotene Bleaching Assay

Evaluation of antioxidants was performed using a method developed by Miller et al. [21]. An aliquot (1 mL) of crystallized β-carotene (0.2 mg/mL in chloroform) was dispensed into a round-bottom flask containing 25 µL of purified linoleic acid and 200 mg of TWEEN 20 emulsifier. The chloroform was removed using a rotary evaporator (Heidolph laborota 4000) and 50 mL of oxygenated, distilled water (shaken at 500 rpm for 30 min) was added to the flask and shaken vigorously. Aliquots (200 µL) of each extract (1 mg/mL) were added to 2.5 mL β-carotene/linoleic acid emulsion by pipetting into a series of spectrophotometer tubes with caps. A zero reading was taken at ƛ_470 nm_ immediately after the addition of the emulsion to the antioxidant solution. Samples were capped and subjected to oxidation by placing in an oven for 3 h at 50 °C. Antioxidant activity was calculated as follows: AA% = (1 − (A_0_ − A_t_/A_00_ − A_0t_)) ×100, where A_0_ is the absorbance at the beginning of the incubation with the extract, A_t_ is the absorbance after 3 h with the extract. A_00_ = is the absorbance at the beginning of the incubation without extract and A_0t_ is the absorbance after 3 h without the extract. Samples were read against a blank containing the emulsion minus β-carotene, i.e., 20 mg linoleic acid with 200 mg TWEEN mixed with 50 mL saturated H_2_O (30 min). BHT and α-tocopherol (1 mg/mL) were tested as comparative positive controls.

#### 2.5.2. ABTS^+^ Radical Scavenging Activity

Radical scavenging activity was determined according to the method first reported by Miller et al. [21] with slight modifications. 2,20-azinobis (3-ethylbenzothiazoline-6-sulfonic acid) (ABTS )was dissolved in water to give a 7 mM concentration stock solution. ABTS radical cations (ABTS^+^) was produced by reacting ABTS stock solution with 2.45 mM potassium persulfate 1:1 (*v*/*v*) and left in the dark at room temperature for 12–16 h before use. ABTS^+^ solution was diluted in 95% ethanol to an absorbance between 0.7 and 0.75 at ƛ_734 nm_. The photometric assay was conducted with 180 µL of the ABTS reagent and 20 µL of the test samples. The optical density was measured at time zero. The radical scavenging activity of the fungal extracts was calculated using the following equation: E = (A_o_ − A_t_)/A_o_ × 100, where A_0_ is the absorbance of the negative control and A_t_ is the absorbance of the samples. Radical scavenging activity was expressed as the concentration that scavenged 50% of the ABTS^+^ radicals (EC_50_). All determinations were carried out in triplicates. Trolox was used as a positive control. The results are normalized and expressed as EC_50_ values (mg extract per mL) for comparison. Effectiveness of antioxidant properties is inversely correlated with EC_50_ value.

#### 2.5.3. Ferric Reducing Antioxidant Power (FRAP)

Reducing power was determined according to the method of Oyaizu [22], with slight modifications. Fungal extracts (200 µL) were mixed with 0.5 mL of 0.2 M phosphate buffer (pH 6.6) and 0.5 mL of 1% (*w*/*v*) potassium ferricyanide. The reaction mixture was incubated at 50 °C for 20 min. After the addition of 0.5 mL of 10% (*w*/*v*) trichloroacetic (TCA), the mixture was centrifuged at 1000 rpm for 10 min using a Heraeus multifuge (DJB labcare Ltd., Buckinghamshire, UK). An aliquot of supernatant (0.5 mL) was mixed with 0.5 mL of deionized water and 0.1 mL of 0.1% (*w*/*v*) ferric chloride. Absorbance was measured at ƛ_700 nm_ against a blank. Higher absorbance indicated greater reducing power. Tests were carried out in triplicates and the results are expressed as mean values ± standard deviations. Reducing power was expressed as the concentration (mg/mL) where the reducing power reached 0.5 (EC_50_). BHT and α-tocopherol were used as positive controls and deionized water was used a negative control. The results are normalized and expressed as EC_50_ values (mg extract per mL) for comparison. Effectiveness of antioxidant properties is inversely correlated with EC_50_ value.

#### 2.5.4. Chelating Effects on Ferrous Ions

The chelating effect on ferrous ions was determined according to the method of Dinis et al. [23]. Each extract (0.5 mL) was mixed with 1.85 mL methanol and 0.05 mL of 2 mM ferrous chloride. The reaction was initiated by the addition of 0.1 mL of 5 mM ferrozine. After 10 min, the absorbance of the mixture was read at ƛ_562 nm_ against a blank. A lower absorbance indicated a higher chelating power. Ethylene diamine tetra acetic acid (EDTA) was used as a standard compound. The effective concentration at which 50% of the ferrous ions were chelated was calculated and obtained by interpolation from linear regression analysis of each extract and EDTA (0.1–50 mg/mL). The chelating activity was expressed as EC_50_, the concentration (mg/mL) that chelated 50% of the Fe^2+^ ions. The results are normalized and expressed as EC_50_ values (mg extract per mL) for comparison. Effectiveness of antioxidant properties is inversely correlated with EC_50_ value.

#### 2.5.5. Cupric Ion Reducing Antioxidant Capacity (CUPRAC)

Total antioxidant potential using Cu (II) as an oxidant was assessed using the CUPRAC assay [1]. To a test tube, 1.5 mL of the test mixture containing 10 mM of copper (II), 7.55 mM neocuproine and 1 M ammonium acetate buffer (pH 7.0) were added. Following this, 0.5 mL of diluted fungal extracts at various concentrations were added to the reaction tubes to achieve a final volume of 2 mL. The tubes were incubated for 30 min at room temperature before the absorbance at ƛ_450 nm_ was recorded against a blank. BHT and α-tocopherol were used as positive controls.

### 2.6. Statistical Analysis

All statistical analyses were conducted using a two-way analysis of variance with Dunnett’s comparison tests or unpaired *t*-tests. These calculations were carried out using GraphPad Prism Software for Windows (GraphPad Software, San Diego, CA, USA). Significance was observed at *p* < 0.05.

## 3. Results

### 3.1. Extraction Yield

Generally, yield and antioxidant activities of extracts rely on the nature of the extracting solvent due to the presence of distinct antioxidant compounds with different chemical characteristics and polarities. In this study, the extraction yields obtained from ethanolic extracts of the mycelium were considerably lower than that of water extracts in both selected media and in all phases of fermentation (Table 1). Significant variations (*p* ≤ 0.05) were also found in the yield of extraction from mycelia of different growth phases. Yields on Day 3 (72 h) were significantly lower (*p* ≤ 0.05) those on Day 5 (120 h) and Day 7 (168 h), whereas no significant differences was found between the yields on Days 5 and 7 as elaborated in Table 1. Highest extraction yields (up to 28.1%) were obtained on Day 5 in MKR media (Table 1). Extraction yields did not change considerably while using Kendrick and Ratledge media (K & R media) throughout the period of fermentation. With respect to strains, MCWJ11 and MC277.49 exhibited higher extraction yields than MC108.16 in MKR media as shown in (Table 1).

#### Antioxidant Components

All major naturally occurring antioxidant classes including polyphenols, flavonoids and condensed tannins were found in each type of extracts. Table 2 summarizes the effect of extraction methods, fermentation period, strains and growth media on the isolation of natural antioxidants. Statistically significant differences (*p* ≤ 0.05) were observed in the extraction of natural antioxidant via ethanol, hot water and crude methods. Ethanolic extraction methodology was found to excel in extraction of all three types of antioxidant components. Noteworthy, the late exponential phase (120 h) appeared to be the optimal time to harvest the cells having maximum quantity of TPCs and TFCs (Table 2). Contrarily, highest amount of condensed tannins were detected during the log phase of growth (Table 2). On the effect of different strains, strain MC277.49 superseded the other two strains significantly (*p* ≤ 0.05) in the production of natural TPCs and TFCs, whereas MC108.16 was found to be the higher producer of condensed tannins in lag phase of growth. Despite the notable difference in the extraction methods, fermentation period and fungal biosynthetic physiology and metabolism, highest antioxidant production was obtained from the cells grown in the nutrient deficient media.

### 3.2. Antioxidant Activity of the Extracts

#### 3.2.1. Total Antioxidant Activity (TOAA)

Total antioxidant activity assay showed significant variations (*p* ≤ 0.05) with respect to different extracts, extraction processes, media, days of fermentations and strains. Crude extracts revealed TOAA greater than ethanolic and aqueous extracts in MKR media (Table 3). Fermentation media also significantly affected (*p* ≤ 0.05) the TOAA capability of selected strains as shown in Table 3. Strains grow on MKR media showed meaningfully higher TOAA than on K & R. Strains MC277.49 and MCWJ11 found to have supreme TOAA on Day 5 of fermentation followed by Day 7.

#### 3.2.2. ABTS Radical Scavenger Activity

The relative radical scavenger activities of each fungal extracts in terms of their EC_50_ are presented in Table 3. Extraction methods showed more obvious variation in this radical scavenging capacity assay of each extract. Unlike TOAA outcomes, ethanolic extracts had highest radical scavenging capacity followed by hot water and crude extracts. Growth period and kind of media also exhibit meaningful differences (*p* ≤ 0.05) on the scavenging capability of antioxidant extracts. Highest radical scavenging activity was shown by the extracts obtained after 120 h (Day 5) of growth in MKR media. MC108.49 and WJ11 are preceded by MC277.49 in the scavenging activity. 

#### 3.2.3. Chelating Ability

The ferrous ion chelating assay measures the capacity of antioxidants to compete with a chelator (2,2-bipyridine or ferrozine) to form chelating complexes with iron (II). In this study, the ability of fungal extracts to slow down or inhibit the complex formation of ferrous ions and ferrozine is analyzed (Table 3). The chelating activity of various fungal extracts at different concentrations were checked and compared to the chelating standard EDTA. The results showed the same trend of antioxidant potential in ABTS and FRAP assays; nonetheless, chelating ability of all extracts were found higher (*p* ≤ 0.05) than the reduction potential. Significant variations (*p* ≤ 0.05) were found in the chelating ability of all extracts with respect to days, extraction process, strains and media. Generally, ethanolic extract showed highest iron binding capability than aqueous and crude extracts, which showed marked variation in the efficiency of different extraction process with respect to target compounds. The highest binding competencies were shown by late exponential growth phase (Day 5) mycelial extracts followed by end phase and lag phase mycelial extracts. Highest chelating power was shown by the ethanolic extracts of strain MC277.49 (1.760 ± 0.16) in late exponential growth phases. Contrarily, the tiniest iron binding ability was represented by the crude extracts of WJ11 (33.517 ± 1.15) taken during initial growth phases in K & R media.

#### 3.2.4. Ferric Reducing Antioxidant Power (FRAP)

FRAP is generally a validated clinical method for calibrating antioxidant potential of plasma [24], but was later extended for its applications to biological systems such as plant, biological fluids, foods, plants extract and now fungal extracts. The measured reducing capacity in FRAP does not necessarily reflect the partial or individual antioxidant activity of components, rather it shows total antioxidant concentration without measuring and summing up the whole antioxidant concentration [24]. The reducing antioxidant power of all extracts is mentioned here in term of EC_50_ (Table 3), whereas reducing potential is inversely proportional to EC_50_. All fungal extracts showed a color change from yellow to purple blue, especially at higher concentration (≥10 mg/mL), in FRAP assay unveiling their reducing potential. The reducing power of all extracts was dose dependent, where ethanolic extracts showed significantly strongest reducing power (*p* ≤ 0.05) as compared to hot water and crude extracts. Despite the different extraction methods adopted, all the extracts showed significant variations (*p* ≤ 0.05) with respect to duration of fermentation and media. Highest reduction potential was seen with five-day-old mycelial extracts (ethanolic) grown in MKR media, whereas the least reduction potential was demonstrated by three-day-old mycelial extracts (especially crude). The strain MC277.49 also showed the highest reducing potential (2.36 ± 0.06) on Day 5 in MKR media, which were comparable to reducing power of standards used, i.e., BHT and α-tocopherol (1.19 ± 0.64). WJ11 exhibited the minimum reducing power (29.19 ± 0.86) on Day 3 in K & R media (especially in crude extracts).

#### 3.2.5. Cupric Ion Reducing Antioxidant Capacity (CUPRAC)

The CUPRAC assay is a variant of FRAP assay that uses copper (Cu) instead of Iron (Fe) [4]. The absorbance of all the fungal extracts and reference compound at concentration (5 mg/mL) was checked at ƛ_450 nm_ and showed in Table 3. Here the data also clearly confirmed our previous findings about the choice of extraction process for the extraction of specific group/class of compounds. Significant differences (*p* ≤ 0.05) were witnessed between the implemented extraction processes. Agitated/refluxed ethanolic extraction was found to be the premier way of extracting the antioxidant which can sequester the metallic ions and hence slowing/preventing the oxidation. Same trend of antioxidant capacity was found in CUPRAC, as described in FRAP, except sequestering power of extracts was detected more in CUPRAC than that of FRAP. Extracts (ethanolic) of strain MC277.49 (4.97 ± 0.31) grown on MKR media were found to possess the highest copper (Cu) sequestering ability on Day 5 when compared to their counterpart strains (Table 3). The least Cu sequestering power was seen in the (crude) extracts of WJ11 (0.9467 ± 0.16) on Day 3 (72 h) grown in K & R media.

## 4. Discussion

Extraction is the first crucial step to obtain natural antioxidants from different sources including filamentous fungi. Many extraction factors such as extraction methods, solvents and their concentrations, time, temperature and pH influence not only the extraction yield but also the effectivity and functionality of resulting extracts. Despite the availability of advanced and non-conventional extraction techniques in the literature, no technique has been specifically addressed for our proposed work; thus, we selected a conventional extraction technique for our studies. However, the adaption of chosen extraction processes provided good yield and insight of antioxidant potentials among crude extracts having intact cell walls; hot water extracts having polysaccharides and other metabolites; and ethanolic extracts rich in polyphenols, flavonoids and condensed tannins. Generally, hot water extraction had higher extraction yield than ethanolic extraction throughout the fermentation media, in all growth media. The credit of highest extraction yield from aqueous extracts goes to soluble polysaccharides, which have higher solubility in polar aqueous solvents such as water [17,25]. This characteristic of polysaccharides is the basis of the hot water extraction of polysaccharide technique, which is only a clinical validated method to dissolve and extract the polysaccharides [26]. Recent studies showed that fungal polysaccharides or their sub-fractions have immune stimulating activity, as well as anti-HIV, anti-herpetic, antiviral, immune regulating and anti-tumor properties [26]. Specifically, aqueous extracts had highest extraction yield in all phases of fermentation in MKR media. The reason of highest extraction from only deficient media (MKR) is that filamentous fungi, including all strains of MC, are involved in the *de-novo* biosynthesis and storage of various metabolites (lipids, GLA, carotenoids, and antibodies) under nitrogen/mineral/nutritional stress and supplemented with excess supply of carbon (C) source(s) [11,16]. The highest extraction yield in both extraction processes and growth media were obtained on the Day 5 of fermentation (Table 1), which is in-line with previous studies [25], suggesting that the maximum production of antioxidants and other metabolites of the fungi is during the late exponential growth phase. Two strains, i.e., MCWJ11 and MC277.49, exhibited higher extraction yield than MC108.9, which may be attributed to their genetic and biochemical variations [14].

Phenolic compounds are a class of natural compounds having functional hydroxyl group (-OH) attached to aromatic hydrocarbon ring. Phenolics are natural lipophilic radical scavengers produced by fungi under the aegis of defense system to cope with different stressful situations. Flavonoids are low molecular weight polyphenolic secondary metabolites having common benzo-γ-pyrone structure and showing enormous biological and pharmacological activities in biological systems [27]. These compounds provide the fungi with natural security against the free radicals, metal chelators and pro-oxidants by acting as reductant, radical scavenger and metallic ion chelator [28,29].

In the present study, TFCs, TPCs and condensed tannins were determined spectrophotometrically according to the recommended methods and calculated as gallic acid equivalent (GAE), quercetin equivalent (QE) and catechin equivalent (CE), respectively. To the best of our knowledge, this is the first comprehensive study reporting the TPCs, TFCs and condensed tannins of important strains of genus *Mucor.* Overall, the fungi contain significant amounts of TPCs, TFCs and condense tannins (Table 2). Ethanolic extracts of all studied strains produced significantly higher yields of polyphenols and flavonoids (*p* ≤ 0.05) than water and crude extracts (Table 2). These findings are similar to previous reports [17,30] stating higher yields of phenols and flavonoids using semi-polar to polar solvents such as ethanol, methanol and water. Therefore, ethanolic extraction procedure has a positive impact on the extraction of phenols and flavonoids followed by hot water extraction (Table 2). Highest TPCs and TFCs were detected in the (ethanolic) extracts of the late exponential growth (Day 5) phases in minimal media, which decreased tremendously (*p* ≤ 0.05) on the Day 7 of fermentation in all strains. On the other hand, condensed tannins were found enormously in the log phase of MC108.49 (5.91 ± 0.65) when grown on MKR media, whereas WJ11 (5.87 ± 0.05) exhibited highest amount of tannins in late exponential phases. Similar amount of condensed tannins was produced in MC108.16 grown in both media, suggesting that its capability to produce tannins is irrespective of the nature of stress. Specifically, MC277.49 was the highest producer of TPCs and TFCs, whereas MC108.16 seemed to produce the greatest surplus of condensed tannins. Therefore, the strains for producing phenolic and flavonoid compounds were in the following order: MC277.49 > MC108.16 > WJ11. However, for condensed tannins, the observed trend was somewhat like this (Table 2): MC108.16 ≥ WJ11 > MC277.49. The difference in the potential of producing varying kinds of bioactive compounds in varying amounts may be attributed to genotypic qualitative and quantitative variations of antioxidant production [14,29] Further, *MC* strains are considered true microbial factories under stress conditions, as they de novo biosynthesize and store various kind of metabolites (lipids, GLA, lycopene, carotenoids, and antibodies) under nitrogen/mineral/nutritional stress conditions provided supplemented with excess supply of carbon (C) source(s) [11,16]. We can deduct from our results (Table 2) that these strains also have the capability of producing higher amount of various secondary metabolites with antioxidant features to cope with unfavorable conditions. The significant reduction of TPCs and TFCs from late exponential phase (120 h) to death phase (168 h) may be due to degradation and consumption of these antioxidant compounds in neutralizing the free radicals, metal ions and pro-oxidants in stress metabolism. Additionally, this study established the facts that all mycelium extracts are good sources of substantial amount of polyphenolic compounds. It is further recognized that further development stages (beyond 120 h) of fungi are not helpful in getting the maximum amount of secondary metabolites, as cited for other higher fungi and mushrooms [31].

TOAA was measured by β-carotene bleaching assay, which is considered a rapid method for antioxidant screening based on the oxidation and discoloration of carotene and linoleic acid by ROSs generated by oxygenated water. Antioxidant activities of all three kinds of extracts (crude, ethanolic and aqueous) of *Mucor circinelloides* were checked and compared with reference standard BHT (butylated hydroxyl toluene) (Table 3). As compared to ethanolic extracts, crude mycelial extracts demonstrated peak TOAAs in protection of linoleic acid and carotene against autoxidation and discoloration. This discrepancy may arise because crude extracts may possess three classes of antioxidant (i.e., enzymes, large molecules such as GLA and proteins; small molecules such as carotene; and higher and lower molecular weight polysaccharides) out of four [32], which imposed a combined effect on inhibition of autoxidation and discoloration of target compounds against generated ROSs. Contrarily, solvents such as ethanol and water targeted only specific classes of antioxidants during extraction [30], hence have less proportion of antioxidants, which in turn showed less TOAA. Periodically, crude extracts from Day 5 showed highest TOAA. The TOAA of all extracts from all phases were in the order of: Day 5 > Day 7 > Day 3. All the extract, with some exceptions, of mycelia grown from nutrient deficient medium showed highest TOAA than complete media. This highest TOAA may be due to higher rate of antioxidant formation in the fungal mycelium under stress conditions, as various authors highlighted a 2.5-fold increase in the formation rate of bioactive components under physiological stress conditions [29]. Furthermore, various ROSs (e.g., H_2_O_2_) generated under these physiological stress conditions acts as signal and transduction molecule affecting/changing/regulating processes such as growth, differentiation, proliferation and expressions of genes, including antioxidant defense (AOD) system genes [29]. Belozerskaya and Gessler, [29] also quoted that any single factor from ROSs (H_2_O_2_ or diamide, decreasing redox potential of the cell) can change the expression of 2499 genes out of 3533 genes of *Aspergellus nidulans*. Deletion of these AOD system genes leads to loss of thermal stability, oxido-reductase activity and make them sensitive to ROSs and other biotic and abiotic stresses [29]. Likewise, MC277.49 (94.15 ± 0.8%) and WJ11 (84.5 ± 1.54%), grown on MKR media and harvested on Day 5 (exponential phase), topped β- carotene bleaching assay. Drakulic et al. [33] also found the same kind of results and documented the maximum intrecellular rodox (E_hc_) potential (–234.99 mV) during exponential phase in yeasts, which lowered to –226.22 mV in stationary phase. The other possibility is that MC277.49 is highest producer of fat-soluble colorant and antioxidants β-carotene and lycopene followed by MC108.16 [13]. With reference to minimal media, many authors also reported variation in media influencing the generation of ROSs in fungi due to which fungi also developed strong AOD system to cope this changes/stresses [34]. Regardless of fact that MC108.16 is a higher β-carotene and lycopene producer [13] than WJ11, WJ11 showed greater TOAA than MC108.19. The reason for higher TOAA of WJ11 might be the higher GLA production, which also possesses antioxidant properties [35]. Finally, from the results, we also concluded that level of ROSs may also act as a switch between different developmental phases of MC as highest ROSs seen in the stationary and death phases of fungi leading to death of cells and these findings also has been published stating the apoptosis of yeasts and other fungi [34].

ABTS^+^ radical scavenging assay is a discoloration assay which measures the capacity of antioxidant to scavenge the ABTS^+^ generated by chemical method. Scavenging occurs through electron donation [17] and here results are adjusted and expressed as EC_50_ relative to positive control Trolox as shown in Table 3. The results of this assay clearly portray the importance of choice in extraction methods for isolation of desired class of antioxidant as ethanolic extracts demonstrated the greatest scavenging activity. The scavenging activity of extraction methods were found to be in descending order of: ethanolic extracts > Hot water extracts > Crude extracts (Table 3). The greater scavenging ability of ethanolic extracts might be due to extraction of more electron donating/hydrogen donating antioxidant by polar organic solvents [30]. As discussed earlier, the radical quenching capability of mycelium also believed to be growth period dependent. Regardless of the strains and extraction methods, the scavenging capacity of five-day-old mycelium extracts showed greater success than Days 7 and 3 mycelia extracts. González-Palma et al. [36] also reported the same results while evaluating the antioxidant activities of filamentous fungi *Pleurotus ostreatus* who noted that antioxidant activities of selected fungi is growth stage dependent and maximum antioxidant activity of methanolic and hot water extracts were shown by 72 h–96 h old extraction samples. Further, the non-variation in our results across three adopted extraction methods and fungal species clearly speaks about the formation and storage of secondary metabolites including radical scavengers till late exponential phase (120 h) under both stress and normal metabolism conditions. The Day 7 mycelial extracts showed slightly less (with some exceptions) radical quenching ability than Day 5, which might be because these secondary metabolites and bioactive components have been used in alternative carbon consuming metabolic pathways or undergo degradation. The three strains of *Mucor* have variable capabilities of *de-novo* biosynthesizing of different storage compounds; the same trends have been seen in their ABTS^+^ scavenging capability, which told us that MC277.49 (3.677 ± 0.45) showed highest quenching ability, followed by MC108.16 (4.183 ± 0.16) and WJ11 (7.6 ± 0.44) on Day 5 under stress conditions (Table 3). The stressful conditions are believed to be responsible for the productions of various extra storage compounds, enzymatic systems and secondary metabolites in filamentous fungi [9,29,36], which partially why all the nutritional deficient media grown filamentous fungi showed higher scavenging ability than normal media. Freimoser and his colleagues [9] also deduced that a change in the stress type and kind of input nutrients also leads to change in the extent of expression of genes responsible for different secondary metabolite production and virulence in Zygomycetes.

Antioxidants may not necessarily show their activity by a direct reaction mechanism. There are numerous other mechanisms exist through which secondary, preventive or type 2 antioxidant my react. These antioxidants do not react directly with free radicals to convert them into stable product but slow down the rate of oxidation by several mechanisms. One of this several mechanisms is chelation of prooxidant metals/transition metals (copper, chromium, cobalt, vanadium, cadmium, arsenic, nickel) which promote the oxidation by acting as catalysts of free radical reactions via donating single electron [4]. Our results (Table 3) demonstrated that the chelating capabilities of all extracts of all under studied strains. The chelating effects of all mycelial extracts on iron increased with increasing concentration. According to their EC_50_ (mg/mL) values, the ethanolic extracts showed highest iron chelating ability than others. This may be due to higher extractability of iron chelators antioxidant via organic solvent systems [37]. However, these findings are not in line with documented chelating ability of different extracts of other filamentous fungi as cited by [17,36] that water extracts were stronger chelating agent than ethanolic extracts. The reasons of this conflict may be attributed to difference in taxonomical groups as filamentous fungi studied so far belong to higher fungi groups called basidiomycetes and ascomycetes whereas MC belongs to lower fungal group called Zygomycetes. The other possible reason is the higher solubility and chelation of metal ions in ethanolic extracts as compared to methanolic extracts. Fungal extracts also showed variations in the intervention of iron and ferrozine complex formation with regards to time period and strains. Highest chelation ability was seen in the 120 h old fungal extracts. Growth period wise, the chelating ability of fungal extracts were found in the order of Late exponential growth phase > Death phase > lag phase (Table 3). Whereas strain wise the chelating ability was found in the descending order of MC277.49 ≥ MC108.16 ≥ WJ11. Highest chelating ability was seen in the (ethanolic) extracts of MC277.49 (1.760 ± 0.16) from MKR media and, on the other hand, least chelating power was shown by the (crude) extracts of WJ11 (33.517 ± 1.15) from the “complete media” (Table 3). The chelating ability of different antioxidant compounds depends upon their internal structure and functional groups used in iron chelation [32,37,38]. The hydroxamate chelators (BHA) showed high iron binding ability whereas the catecholate chelators (CA and DHBA) showed low iron binding ability. Similarly, Desferroxamine B had the highest iron binding ability due to the three biden-tate ligand structures within the molecule. Roosenberg et al. [38] also reported the highest effectiveness of siderophores is due to three bidentate ligands inside their structure that enable them to form hexaden-tate complexes with smaller entropic variations than that caused by chelating one ferric ion with separate ligands. The variations in the chelation power of different extracts with respect of strains, time growth, and media may infer existence and manifestation of different antioxidants at different concentration in different strains at different time intervals with possible variations in their mechanisms. As described earlier, zygomycetes can produce a wide range of metabolites and commercially important compounds [10] this investigation also proved that MC biomass or further processed extracts are also a good source of iron chelator antioxidants which show proven evidences of slowing down the rate of pro-oxidant metals. Ferrous ions are the most active pro-oxidants/catalysts in the food systems especially in fat and oils for the initial formation of reactive oxygen species. The fungal ethanolic extracts might also be considered to impart in various food formulations as cheap natural metal chelators.

FRAP is robust, reliable, inexpensive antioxidant assay which can be used for testing of both single or mixture of antioxidants in aqueous solution via automated, semi-automated and manual methods. It is also discoloration assay which measures the reducing power of potential antioxidants (reductants) by reducing Fe^3+^ to Fe^2+^ (oxidant), causing a change in color measurable at 593 nm [32]. In this study, the antioxidant potentials of the fungi were measured by hydrogen donating capability of their antioxidants. As discussed in results, each fungus possessed the reducing power and this reducing power markedly differ (*p* ≤ 0.05) with respect to ethanolic, aqueous and crude extracts, growth period and strain. Generally, ethanolic extracts showed strongest reducing power than hot water and crude extracts. The reason for variation in these outcomes may be due to the higher extractability of hydrogen donating antioxidants via organic solvents such as ethanol and methanol. The ethanolic extraction found to be in a positive relationship with the extraction of hydrogen donating bio-actives/antioxidants and differ significantly (*p* ≤ 0.05) from hot water and crude extracts. Notably, the fungal extracts also showed significant variation (*p* ≤ 0.05) with respect to growth phases. Highest reducing power was exhibited by the extracts taken from log and stationary phases (120 h), during which fungal strains form and stored the various storage compounds and secondary metabolites for survival and to cope with the unfavorable conditions in death phase [28]. The outcomes of FRAP assay are generally in common with the ABTS assay results, which also revealed a relationship between these two methods. The comparable redox potential of ABTS^+^ (0.68 V) and Fe^3+^, and working principle (i.e., electron/hydrogen donating potential) [17,32] are probably the main causative factors for their close association in results. The ethanolic, aqueous and crude extracts of MC277.49 were found to be stronger reducing agent compared to other strains. Specifically, the ethanolic extract of MC277.49 from exponential phase had the lowest effective concentration (2.36 ± 0.06) at which the absorbance was 0.5 for reducing power (Table 3) followed by MC108.16 (3.68 ± 0.06) and WJ11 (5.540 ± 0.06). The reducing power of the same strains reduced slightly both on Day 7 and in hot water extracts, which might be due to the degradation of reductant antioxidants compounds and sufficient inefficiency of water solvent system to extract antioxidants compounds respectively. It is evident here that fungal extract showed a dose related effect. At higher concentrations (≥10 mg/mL), the reducing power of these extracts (ethanolic) were similar to those of the standards used, i.e., BHT and α-tocopherol. At the concentration of 50 mg/mL, the reducing power of ethanolic extracts of MC2777.49 was strongest, which may be attributed to highest contents of polyphenols and reductones [32]. The reducing potential also detected to be stress and species specific, and this variation in reducing power may be due to variation in the genetic capability to produce antioxidants or due to production of that kind of antioxidants which are unable to reduced Fe^3+^ by hydroxylation and conjugation such as thiol antioxidants (e.g., glutathiol) [17,32]. Our findings are also in agreement with Emri et al. [28], who llustrated the higher production of secondary metabolites and enzymatic antioxidants under stress conditions as result of Cross-stress protection phenomena. This cross-stress protection phenomenon based on cluster of genes called “Environmental Stress Response” or ESR genes that have been identified in *S. cerevisiae, S. pombe, Candida albicans, Candida glabrata, Aspergillus fumigatus, Methanosarcina barkeri* and in other *Ascomycetes*, for example, *Claviceps purpurea, Neuropsora crassa, Magnaporthe oryzae*, and *Fusarium graminearu* [28]. This stress conditions has been found to up-regulate the ESR genes in many filamentous fungi. These stresses based genome-wide transcriptional changes are depended on the type and strength of the stress.

The CUPRAC assay was initially introduced by Apak et al. [4] and has also been successfully applied to antioxidants in food plants, human serum, and to hydroxyl radical scavengers. This method is more stable, rapid, selective and suitable for both hydrophilic and lipophilic antioxidants [4]. It employed copper (II)–neocuproine (Cu(II)–Nc) reagent as a chromogenic oxidizing agent. The application of CUPRAC in this study contributed to the evaluation of antioxidant potential of these species. This is the first report on the appraisal of antioxidant activity of the mycelium of these fungal strains, using these extraction techniques tested by the CUPRAC method. At 5 mg/mL, ethanolic extracts showed highest Cu sequestering activity than other kind of extracts of all fungal extracts. The highest sequestering and reducing activity of ethanolic extracts depicted the higher solubility and hence the extraction potential of Cu sequestering and reducing antioxidants using ethanol in solvent extraction. Hot water extracts came after the ethanolic extracts in extractability of Cu sequestering and reducing antioxidants followed by crude extracts. Additionally, ethanolic extracts which were statistically significant (*p* ≤ 0.05) from both BHT and α-tocopherol also showed lowest EC_50_ values (*p* ≤ 0.05) for FRAP (Table 3). This relationship between different antioxidant assays suggests that the antioxidant capacity is not merely a response of flavonoids or polyphenols but also depends upon the electron donating ability. The ethanolic extracts (5 mg/mL) of all the strains and period of growth showed higher Cu sequestering capabilities than the reference compounds (1 mg/mL), i.e., BHT and α-tocopherol (Table 3). The ethanolic extracts of strain CBS277.49 (4.97 ± 0.31) were found to possess more than double antioxidant capacity as compared to the standard’s reducing potential. Irrespective of the extraction process and media employed, the Cu sequestering ability of all strains was found in the descending order of: MC277.49 > MC 108.16 > WJ11. The minimum Cu reducing potential was seen in the lag phase of WJ11 (0.9467 ± 0.16) on MK media (Table 3). With respect to hydrophobic and hydrophilic antioxidant capacity measuring assays (i.e., ABTS and CUPRAC), ethanolic and hot water extracts of MC277.49 were found to be most effective as compared to crude extracts. The possible relationship behind this is due to radical scavenging ability through electron transfer. Overall, the extracts of all strains from late exponential growth phases, grown on minimal media, were found strong reductants, metal chelators and radical scavengers.

### Correlation of Antioxidant Components and Antioxidant Activities

Correlation analysis was used to explore the relationship between the antioxidant components and antioxidant capacity assays (ABTS, FRAP, CUPRAC, Iron chelating and β-carotene assays) measured for all extracts from three strains of *MC*. All strains were found to be rich producers of TPCs followed by condensed tannins and TFCs (Table 2). An obvious and powerful relation was observed for TPCs and all other calorimetric assays. TPCs extracted by ethanol, hot water and crude methods showed the highest association with the ABTS, FRAP and CUPRAC assays (Table 3). Highest (ethanolic) phenolic contents were obtained in the late exponential phases (120 h) of all strains via ethanolic extraction way. It is also evident from the results (Table 3) that these highest phenolic contents in late exponential phases (Table 2) contributed significantly (*p* ≤ 0.05) positively towards the ABTS^+^ scavenging ability, metal ion chelation and reducing power potential in the same period for the majority of strains. This strong correlation demonstrated that these compounds are responsible for the major antioxidant activities. Highest antioxidant capacity shown by ethanolic extracts revealed that different compounds are extracted using different extraction solvent systems and extraction procedures and these different extracted components in turn have different affinities for different oxidative processes due to their varied polarities. Despite of the fact that crude extracts have been shown to possess significantly (*p* ≤ 0.05) lower amounts of TPCs, TFCs and condensed tannins, these extracts showed excellent antioxidant activities via protecting carotene and linoleic acid against discoloration and oxidation by ROSs. This inconsistency may arise because crude biomass of all strains is considered a rich source of reducing agents, proteins, peptides, amino acids, phytosterols, nucleotides microelements, carotenoids, lycopene and organic acids, which also play impart their role in protecting linoleic acid and carotene, besides antioxidants [39]. Some reports solely underline the potential interactions and synergic effects of these components in enhancing the antioxidant potentials of extracts [39]. Additionally, the chelating ability of hot water extracts and crude extracts (Table 3) may also be due to various water-soluble components, e.g., lower molecular weight polysaccharides, which have been reported to possess antioxidant activity [25]. The increasing potential of antioxidants is synchronized with increased productions of colored pigments β-carotene and lycopene in late exponential phase. These results are also believed to be due to higher production of secondary metabolites of filamentous fungi in the form of phenols, flavonoids and other colored pigments. Previous works have also reported the successful highest production of natural colorants, i.e., β-carotene and lycopene, from *MC* strains under nutritional stress conditions in late exponential growth phases. These natural colorants also have antioxidant activity, and it is possible that variation in the antioxidant potential of these strains is attributable to the variation in their genetic capacities of producing these colorants, storage compounds and secondary metabolites. It is also worth noting that condensed tannins were detected in greater amount during lag growth phase of MC108.16 and amount of condensed tannins were also found greater than flavonoids in all strains throughout the specified life span. These findings disclosed the dynamic approach and positive dependency of all strains not only on phenolic compounds but also on condensed tannins in formation of their strong defense system.

Finally, phenols, condensed tannins and flavonoids were found to be major effective antioxidant components in different in vitro assays showing satisfactory and comparable results to reference compounds (Table 2). Based on all of our above stated results, it is established that all fungal extracts showed great potential in prevention of oxidation by preventing/slowing down radical formation, and as ion chelators and pro-oxidants in all biological systems including food and pharmaceuticals systems. These findings suggest the potential use of these fungal extracts as an additive in the food and pharmaceutical industries.

## 5. Conclusions

The present investigation clearly depicts the fungal mycelium as rich source of antioxidants and other secondary metabolites. All kind of fungal extracts showed excellent radical scavenging, reducing and metal chelating properties in ABTS, FRAP, CUPRAC, β-carotene, and iron chelating assays. The antioxidant recovery yield from fungal mycelium differed, and depended on the solvent system and extraction process. Highest recovery yield was obtained in hot water extraction process and ethanolic extracts were found to be most effective with reference to antioxidant properties. It was found that the overall antioxidant property of all extracts of all strains were attributable to their phenolic and condensed tannin contents. Strains MC277.49 was found to be the biggest producer of secondary metabolites under nutritional stress condition in late exponential phase. These *Mucor* strains prove to be rich sources of antioxidants and secondary metabolites, which could be used in the development of nutraceuticals and natural antioxidants.

## Figures and Tables

**Table 1:** Extraction yield (%) from all fungal strains following the two extraction methods.

Selected Fungal Strains	Days	% Recovery *			
		Ethanolic Extracts		Water Extracts	
		MKR ** Media	K&R *** Media	MKR Media	K&R Media
*Mucor circinelloides* (WJ11)	3	9.68 ± 0.94 ^h^	6.62 ± 0.94 ^e^	23.2 ± 1.06	22.18 ± 1.68
*Mucor circinelloides* (CBS 277.49)		11.6 ± 0.66	8.47 ± 0.73 ^e^	22.40 ± 0.49	22.6 ± 1.32
*Mucor circinelloides* (CBS 108.16)		11.48 ± 0.94	7.69 ± 0.64 ^e^	20.74 ± 1.65 ^d^	21.58 ± 1.47 ^d^
*Mucor circinelloides* (WJ11)	5	16.69 ± 1.17 ^e^	9.24 ± 0.9 ^e^	28.16 ± 1.65 ^a^	23.28 ± 0.93
*Mucor circinelloides* (CBS 277.49)		15.68 ± 1.12 ^e^	9.40 ± 0.72 ^e^	27.35 ± 1.72 ^a^	25.30 ± 1.73
*Mucor circinelloides* (CBS 108.16)		15.27 ± 0.73	9.26 ± 0.67 ^e^	26.88 ± 0.76	26.65 ± 1.39 ^a^
*Mucor circinelloides* (WJ11)	7	16.55 ± 1.13 ^e^	8.63 ± 0.77 ^e^	27.56 ± 1.56 ^a^	24.35 ± 1.24
*Mucor circinelloides* (CBS 277.49)		15.1 ± 1.27	9.13 ± 1.2 ^e^	26.45 ± 1.74	25.70 ± 1.26
*Mucor circinelloides* (CBS 108.16)		15.22 ± 0.85	8.63 ± 0.29 ^e^	25.76 ± 0.93	23.55 ± 1.17

Note: * Total dry weight (%) recovered following extraction; ** Modified Kendirck and Ratledge media; *** Kendrick and Ratledge media. Data represent the mean ± SD (*n* = 3) of recovered dry extract following hot water and methanol extraction from crude mycelial biomass (5%, *w*/*v*). For the different fungi, with different growth periods and media, means with different letters were significantly different, with alphabetical order of letters (a–h) representative of the highest to lowest content. The values without a letter are considered statically non-significant.

**Table 2:** Summary of antioxidant components found periodically in each selected strain in their three extracts.

Days	Extraction Methods	AOX. Components	MC-WJ11		MC-CBS 277.49		MC-CBS 108.16	
			K&R Media	MKR Media	K&R Media	MKR Media	K&R Media	MKR Media
3	EtOh Extracts	TPC * _(mg GAE/g of dry extract)_	11.31 ± 0.74	14.7 ± 0.1	17.7 ± 0.48	25.6 ± 0.75 ^b^	16.62 ± 0.6	22.41 ± 0.72
		TFC ** _(mg QE/g of dry extract)_	0.13 ± 0.01	0.19 ± 0.01	0.10 ± 0.0	0.17 ± 0.0	0.06 ± 0.0	0.07 ± 0.0
		Tannins *** _(mg CE/g of dry extract)_	3.38 ± 0.56	3.93 ± 0.27	3.91 ± 0.09	4.5 ± 0.09 ^b^	5.82 ± 0.24 ^a^	5.91 ± 0.65 ^a^
	Aqueous Extracts	TPC * _(mg GAE/g of dry extract)_	8.67 ± 0.48	9.83 ± 0.34	13.3 ± 0.7	18.2 ± 0.59	10.02 ± 0.03	14.27 ± 0.15
		TFC ** _(mg QE/g of dry extract)_	0.10 ± 0.0	0.12 ± 0.02	0.07 ± 0.01	0.13 ± 0.02	0.04 ± 0.0	0.053 ± 0.2
		Tannins *** _(mg CE/g of dry extract)_	1.02 ± 0.11	2.24 ± 0.03	2.71 ± 0.08	3.34 ± 0.1	4.01 ± 0.1	4.2 ± 0.1
	Crude Extracts	TPC * _(mg GAE/g of dry extract)_	2.20 ± 0.08	5.31 ± 0.2	3.83 ± 0.07	8.54 ± 0.08	4.41 ± 0.1	6.6 ± 0.11
		TFC ** _(mg QE/g of dry extract)_	0.08 ± 0.01	0.09 ± 0.0	0.02 ± 0.0	0.06 ± 0.01	0.02 ± 0.0	0.03 ± 0.0
		Tannins *** _(mg CE/g of dry extract)_	0.91 ± 0.07	1.14 ± 0.03	1.95 ± 0.05	2.6 ± 0.17	1.36 ± 0.12	1.97 ± 0.12
5	EtOh Extracts	TPC * _(mg GAE/g of dry extract)_	16.34 ± 0.4	18.75 ± 0.57	23.56 ± 0.06	27.26 ± 0.49 ^a^	19.4 ± 0.48	24.39 ± 0.66
		TFC ** _(mg QE/g of dry extract)_	0.018 ± 0.00	0.17 ± 0.0	0.27 ± 0.01	0.31 ± 0.01 ^a^	0.16 ± 0.0	0.27 ± 0.01
		Tannins *** _(mg CE/g of dry extract)_	3.59 ± 0.3	5.87 ± 0.05 ^a^	3.08 ± 0.05	3.64 ± 0.12	2.52 ± 0.03	2.94 ± 0.05
	Aqueous Extracts	TPC * _(mg GAE/g of dry extract)_	13.42 ± 0.13	15.49 ± 0.6	19.23 ± 0.4	23.7 ± 0.45	15.06 ± 0.5	21.1 ± 0.51
		TFC ** _(mg QE/g of dry extract)_	0.01 ± 0.0	0.13 ± 0.01	0.19 ± 0.00	0.31 ± 0.03 ^a^	0.12 ± 0.0	0.24 ± 0.06
		Tannins *** _(mg CE/g of dry extract)_	2.19 ± 0.1	4.46 ± 0.65 ^b^	2.55 ± 0.48	2.89 ± 0.2	1.66 ± 0.41	2.25 ± 0.27
	Crude Extracts	TPC * _(mg GAE/g of dry extract)_	7.64 ± 0.73 ^a^	11.75 ± 0.6	16.75 ± 0.57	16.71 ± 0.1	13.33 ± 0.12	15.1 ± 0.11
		TFC ** _(mg QE/g of dry extract)_	0.005 ± 0.0 ^x^	0.01 ± 0.0	0.08 ± 0.01	0.11 ± 0.02	0.05 ± 0.0	0.08 ± 0.01
		Tannins*** _(mg CE/g of dry extract)_	1.84 ± 0.07	2.35 ± 0.01	1.8 ± 0.1	2.05 ± 0.03	0.92 ± 0.02	1.16 ± 0.02
7	EtOh Extracts	TPC * _(mg GAE/g of dry extract)_	3.29 ± 0.09	8.50 ± 0.54	20.3 ± 0.64	23.46 ± 0.9	13.19 ± 0.24	17.5 ± 0.28
		TFC ** _(mg QE/g of dry extract)_	0.07 ± 0.00	0.13 ± 0.05	0.18 ± 0.01	0.24 ± 0.01	0.08 ± 0.01	0.22 ± 0.04
		Tannins *** _(mg CE/g of dry extract)_	1.88 ± 0.21	3.23 ± 0.06	2.78 ± 0.33	3.51 ± 0.62	1.68 ± 0.14	2.2 ± 0.07
	Aqueous Extracts	TPC * _(mg GAE/g of dry extract)_	2.43 ± 0.23	6.02 ± 0.39	17.09 ± 0.2	21.4 ± 0.59	8.89 ± 0.12	15.8 ± 0.40
		TFC ** _(mg QE/g of dry extract)_	0.056 ± 0.01	0.08 ± 0.01	0.16 ± 0.00	0.18 ± 0.01	0.05 ± 0.01	0.16 ± 0.01
		Tannins *** _(mg CE/g of dry extract)_	1.6 ± 0.2	2.32 ± 0.01	2.62 ± 0.24	3.1 ± 0.46	1.2 ± 0.04	1.88 ± 0.08
	Crude Extracts	TPC * _(mg GAE/g of dry extract)_	1.12 ± 0.1 ^h^	3.83 ± 0.2	9.14 ± 0.08	13.6 ± 0.45	4.72 ± 0.55	8.6 ± 0.39
		TFC ** _(mg QE/g of dry extract)_	0.01 ± 0.0	0.03 ± 0.0	0.02 ± 0.0	0.06 ± 0.0	0.02 ± 0.00	0.07 ± 0.02
		Tannins *** _(mg CE/g of dry extract)_	0.71 ± 0.04 ^x^	0.93 ± 0.31	0.8 ± 0.04 ^wx^	1.2 ± 0.02	0.7 ± 0.01 ^x^	1.02 ± 0.13

Note: Results displayed are a representation of triplicate quantifications per extract. * Total phenolic content based on calibration curve of gallic acid, expressed as mg gallic acid equivalents (GAE) per g of dry extract; *r* = 0.993. ** Flavonoid content expressed as mg quercetin equivalents (QE) per g of dry extract; *r* = 0.9891. *** Total condensed tannin content based on calibration curve of (+)-catechin, expressed as mg catechin equivalents (CE) per g of dry extract; *r* = 0.991. The Dunnett’s test was to evaluate the significance of compound isolation following hot water and methanol extraction in comparison to the crude extract, confidence level was set to 95%. For the different fungi, with different growth periods and media, means with different letters were significantly different, with alphabetical order of letters (a–x) representative of the highest to lowest content. The values without a letter are considered statically non-significant.

**Table 3:** Summary of antioxidant assays of three types of fungal mycelia extracts grown on two kinds of media and harvested at different growth phases.

Days	Extraction Methods	AOX. Assays	MC-WJ11		MC-CBS 277.49		MC-CBS 108.16		Controls
			K&R Media	MKR Media	K&R Media	MKR Media	K&R Media	MKR Media	
3	EtOh Extracts	β-Carotene	44.4 ± 2.15	52.86 ± 1.07 ^q^	67.2 ± 1.09	73.90 ± 0.55	63.32 ± 0.47	67.5 ± 1.38	77.08 ± 2.2 ^+^
		ABTS ^a^	13.27 ± 1.06	11.58 ± 0.44	7.65 ± 0.15	5.60 ± 0.09	8.54 ± 0.43	8.65 ± 0.22	1.74 ± 0.3 ^++^
		Chelating ^b^	11.15 ± 0.14	10.08 ± 0.12	6.97 ± 0.43	4.64 ± 0.1	9.32 ± 0.17	7.32 ± 0.16	1.8 ± 0.45 ^+++^
		Reducing Power ^c^	13.69 ± 0.24	9.37 ± 0.25	7.16 ± 0.07	5.02 ± 0.13 ^a^	8.14 ± 0.11	7.37 ± 0.07	1.19 ± 0.64 ^++++^
		CUPRAC	2.09 ± 0.06	3.13 ± 0.13	2.86 ± 0.17	4.33 ± 0.38 ^b^	2.12 ± 0.05	2.64 ± 0.11	2.19 ± 0.04 ^+^
	Aqueous Extracts	β-Carotene	26.94 ± 0.15 ^z^	34.45 ± 0.17 ^y^	43.30 ± 0.07	51.14 ± 0.08	42.77 ± 0.31	42.7 ± 0.15	77.08 ± 2.2 ^+^
		ABTS ^a^	15.5 ± 0.29	12.46 ± 0.36	8.80 ± 0.15	7.33 ± 0.15	10.07 ± 0.10	10.22 ± 0.49	1.74 ± 0.3 ^++^
		Chelating ^b^	14.93 ± 0.82	13.34 ± 0.53	9.19 ± 0.34	6.26 ± 0.17	11.74 ± 0.25	9.12 ± 0.19	1.8 ± 0.45 ^+++^
		Reducing Power ^c^	18.5 ± 0.33	15.23 ± 0.46	11.30 ± 0.30°	7.96 ± 0.16	14.54 ± 0.72	10.15 ± 0.08	1.19 ± 0.64 ^++++^
		CUPRAC	1.22 ± 0.17	1.45 ± 0.16	1.8 ± 0.04	2.23 ± 0.04	1.49 ± 0.11	1.91 ± 0.08	2.19 ± 0.04 ^+^
	Crude Extracts	β-Carotene	58.58 ± 1.24 ^p^	68.5 ± 0.66	76.71 ± 1.34	81.37 ± 1.59	71.5 ± 1.69	74.22 ± 1.19	77.08 ± 2.2 ^+^
		ABTS ^a^	24.32 ± 0.24 ^a^	22.09 ± 0.25 ^b^	17.64 ± 0.26 ^d^	13.36 ± 0.16	21.22 ± 0.60 ^b^	15.75 ± 0.32	1.74 ± 0.3 ^++^
		Chelating ^b^	33.51 ± 1.15 ^a^	27.84 ± 0.27 ^b^	13.88 ± 0.60	11.65 ± 0.15	24.4 ± 0.70 ^c^	18.26 ± 0.12 ^e^	1.8 ± 0.45 ^+++^
		Reducing Power ^c^	29.19 ± 0.86 ^a^	21.34 ± 0.25 ^c^	18.13 ± 0.22	14.16 ± 0.09	23.17 ± 0.62 ^b^	19.38 ± 0.13	1.19 ± 0.64 ^++++^
		CUPRAC	0.94 ± 0.16 ^y^	1.03 ± 0.08	1.44 ± 0.05	1.77 ± 0.05	1.13 ± 0.05	1.31 ± 0.05	2.19 ± 0.04 ^+^
5	EtOh Extracts	β-Carotene	63.96 ± 0.53	73.23 ± 1.02	74.18 ± 1.3	79.67 ± 1.2	66.6 ± 1.34	63.86 ± 1.52	77.08 ± 2.2 ^+^
		ABTS ^a^	9.22 ± 0.59	7.6 ± 0.44	5.47 ± 0.18	3.67 ± 0.45 ^g^	6.66 ± 0.26	4.18 ± 0.16	1.74 ± 0.3 ^++^
		Chelating ^b^	6.43 ± 0.09	4.70 ± 0.36	2.6 ± 0.1	1.76 ± 0.16 ^b^	5.09 ± 0.19	3.96 ± 0.15	1.8 ± 0.45 ^+++^
		Reducing Power ^c^	11.04 ± 0.24	5.54 ± 0.06	4.73 ± 0.16	2.36 ± 0.06 ^d^	8.47 ± 0.1	3.68 ± 0.06	1.19 ± 0.64 ^++++^
		CUPRAC	2.07 ± 0.05	3.16 ± 0.05	3.23 ± 0.22	4.97 ± 0.31 ^a^	2.41 ± 0.30	3.43 ± 0.23 ^c^	2.19 ± 0.04 ^+^
	Aqueous Extracts	β-Carotene	39.22 ± 0.07 ^x^	47.58 ± 0.68	49.04 ± 0.73	51.31 ± 0.73	42.40 ± 0.72	42.4 ± 0.74	77.08 ± 2.2 ^+^
		ABTS ^a^	10.68 ± 0.79	9.24 ± 0.57	6.16 ± 0.54	4.72 ± 0.22	8.13 ± 0.3	6.11 ± 0.10	1.74 ± 0.3 ^++^
		Chelating ^b^	8.23 ± 0.17	5.26 ± 0.14	3.25 ± 0.10	2.95 ± 0.14	4.20 ± 0.12	6.23 ± 0.12	1.8 ± 0.45 ^+++^
		Reducing Power ^c^	13.30 ± 0.20	8.42 ± 0.3	6.53 ± 0.34	3.9 ± 0.22	10.65 ± 0.33	4.51 ± 0.16	1.19 ± 0.64 ^++++^
		CUPRAC	1.41 ± 0.06	1.78 ± 0.08	2.06 ± 0.11	3.32 ± 0.03	1.65 ± 0.05	2.68 ± 0.03	2.19 ± 0.04 ^+^
	Crude Extracts	β-Carotene	69.67 ± 1.53	84.5 ± 1.54 ^c^	83.3 ± 1.04	94.15 ± 0.85 ^a^	77.1 ± 1.45	73.1 ± 1.21	77.08 ± 2.2 ^+^
		ABTS ^a^	16.80 ± 0.15	14.23 ± 0.23	10.45 ± 0.23	7.96 ± 0.27	14.18 ± 0.08	13.13 ± 0.56	1.74 ± 0.3 ^++^
		Chelating ^b^	24.35 ± 0.14 ^c^	15.41 ± 0.31 f	11.47 ± 0.1	7.43 ± 0.09	15.36 ± 3.50 ^f^	12.25 ± 0.11	1.8 ± 0.45 ^+++^
		Reducing Power ^c^	17.82 ± 0.14	11.41 ± 0.11°	12.69 ± 0.09	5.46 ± 0.46	16.45 ± 0.27 ^h^	9.63 ± 0.46	1.19 ± 0.64 ^++++^
		CUPRAC	1.19 ± 0.01	1.34 ± 0.04	1.69 ± 0.03	2.88 ± 0.09	1.31 ± 0.11	2.14 ± 0.05	2.19 ± 0.04 ^+^
7	EtOh Extracts	β-Carotene	61.7 ± 1.34	71.57 ± 0.86	71.81 ± 1.41	74.75 ± 0.0	71.81 ± 2.08	61.92 ± 0.57	77.08 ± 2.2 ^+^
		ABTS ^a^	9.92 ± 0.23	9.6 ± 0.66	4.51 ± 0.21	5.22 ± 0.07	7.38 ± 0.35	7.7 ± 0.06	1.74 ± 0.3 ^++^
		Chelating ^b^	7.12 ± 0.04	5.29 ± 0.15	4.24 ± 0.19	2.43 ± 0.06	6.06 ± 0.20	4.81 ± 0.15	1.8 ± 0.45 ^+++^
		Reducing Power ^c^	10.18 ± 0.11	6.67 ± 0.21	7.66 ± 0.13	3.11 ± 0.11	9.16 ± 0.34	5.27 ± 0.21 ^a^	1.19 ± 0.64 ^++++^
		CUPRAC	1.68 ± 0.08	2.76 ± 0.15	2.92 ± 0.14	4.01 ± 0.12 ^b^	1.88 ± 0.08	3.063 ± 0.24	2.19 ± 0.04 ^+^
	Aqueous Extracts	β-Carotene	44.47 ± 1.15	46.25 ± 0.39	49.72 ± 0.64	50.54 ± 1.14	33.9 ± 1.10 ^y^	40.3 ± 0.73	77.08 ± 2.2 ^+^
		ABTS ^a^	11.31 ± 0.43	10.81 ± 0.47	5.66 ± 0.15	7.043 ± 0.58	8.36 ± 0.23	9.07 ± 0.40	1.74 ± 0.3 ^++^
		Chelating ^b^	9.21 ± 0.10	6.32 ± 0.53	6.07 ± 0.25	4.12 ± 0.37	8.26 ± 0.26	5.67 ± 0.37	1.8 ± 0.45 ^+++^
		Reducing Power ^c^	13.6 ± 0.67	8.02 ± 0.23	9.46 ± 0.38	5.22 ± 0.18 ^a^	11.7 ± 0.29	6.73 ± 0.35	1.19 ± 0.64 ^++++^
		CUPRAC	1.32 ± 0.05	1.62 ± 0.06	1.88 ± 0.05	2.813 ± 0.06	1.42 ± 0.02	1.91 ± 0.02	2.19 ± 0.04 ^+^
	Crude Extracts	β-Carotene	65.8 ± 1.44	81.23 ± 1.97	80.21 ± 1.12	89.02 ± 1.03 ^b^	64.47 ± 1.09	72.29 ± 1.12	77.08 ± 2.2 ^+^
		ABTS ^a^	19.26 ± 0.15 ^c^	17.3 ± 0.46 ^d^	12.07 ± 0.61	12.12 ± 0.61	16.77 ± 0.97	14.33 ± 0.56	1.74 ± 0.3 ^++^
		Chelating ^b^	19.83 ± 0.09	21.6 ± 0.20 ^d^	13.17 ± 0.1	9.47 ± 0.41	18.42 ± 0.12 ^e^	14.44 ± 0.24	1.8 ± 0.45 ^+++^
		Reducing Power ^c^	19.68 ± 0.71 ^d^	17.06 ± 0.23	14.59 ± 0.38	8.10 ± 0.10	17.75 ± 0.22	15.32 ± 0.44 ^I^	1.19 ± 0.64 ^++++^
		CUPRAC	1.1 ± 0.04	1.25 ± 0.06	1.56 ± 0.06	2.29 ± 0.01	1.16 ± 0.01	1.443 ± 0.03	2.19 ± 0.04 ^+^

Note: β-carotene of selected fungal extracts (5 mg/mL) based on percent bleaching inhibition. CUPRAC of selected fungal extracts (10 mg/mL) based on absorbance (ƛ450 nm). EC_50_ values ^a,b,c^ (mg/mL) of fungal extracts in terms of ABTS^+^ scavenging, reducing power and chelating ability assays, data are expressed as means ± SD (*n* = 3) of upper and lower limit of dose interpolation curve, with 95% confidence. ^a^ EC_50_ (mg/mL) is representative of the effective concentration at which 50% of ABTS^+^ radicals were scavenged. ^b^ EC_50_ (mg/mL) is representative of the effective concentration at which 50% of Fe^2+^ ions were chelated. ^c^ EC_50_ (mg/mL) is representative of the effective concentration at which the absorbance was 0.5. Controlled: ^+^ BHT; ^++^ Trolox, ^+++^ α-tocopherol; ^++++^ EDTA. Significant correlation of antioxidant activity in terms of ABTS^+^ activity, reducing power and chelating ability to phenolic, flavonoid and condensed tannin content denoted by x, y and z, where confidence level was set to 95%, respectively. For the different fungi, with different growth periods and media, means with different letters were significantly different, with an alphabetical order of letters (a–z) representative of the highest to lowest contents. The values without a letter are considered statically non-significant.
